# RTUAV-YOLO: A Family of Efficient and Lightweight Models for Real-Time Object Detection in UAV Aerial Imagery

**DOI:** 10.3390/s25216573

**Published:** 2025-10-25

**Authors:** Ruizhi Zhang, Jinghua Hou, Le Li, Ke Zhang, Li Zhao, Shuo Gao

**Affiliations:** 1 United Laboratories of TT&C and Communication, Jiuquan Satellite Launch Center, Lanzhou 732750, China; zhangruizhi1996@buaa.edu.cn (R.Z.); hjh20jd@163.com (J.H.); lile501@163.com (L.L.); kekedou202508@163.com (K.Z.); 13311123825@163.com (L.Z.); 2 School of Instrumentation and Optoelectronic Engineering, Beihang University, Beijing 100191, China

**Keywords:** RTUAV-YOLO, small objects detection, UAV aerial imagery, lightweight model, YOLOv11

## Abstract

Real-time object detection in Unmanned Aerial Vehicle (UAV) imagery is critical yet challenging, requiring high accuracy amidst complex scenes with multi-scale and small objects, under stringent onboard computational constraints. While existing methods struggle to balance accuracy and efficiency, we propose RTUAV-YOLO, a family of lightweight models based on YOLOv11 tailored for UAV real-time object detection. First, to mitigate the feature imbalance and progressive information degradation of small objects in current architectures multi-scale processing, we developed a Multi-Scale Feature Adaptive Modulation module (MSFAM) that enhances small-target feature extraction capabilities through adaptive weight generation mechanisms and dual-pathway heterogeneous feature aggregation. Second, to overcome the limitations in contextual information acquisition exhibited by current architectures in complex scene analysis, we propose a Progressive Dilated Separable Convolution Module (PDSCM) that achieves effective aggregation of multi-scale target contextual information through continuous receptive field expansion. Third, to preserve fine-grained spatial information of small objects during feature map downsampling operations, we engineered a Lightweight DownSampling Module (LDSM) to replace the traditional convolutional module. Finally, to rectify the insensitivity of current Intersection over Union (IoU) metrics toward small objects, we introduce the Minimum Point Distance Wise IoU (MPDWIoU) loss function, which enhances small-target localization precision through the integration of distance-aware penalty terms and adaptive weighting mechanisms. Comprehensive experiments on the VisDrone2019 dataset show that RTUAV-YOLO achieves an average improvement of 3.4% and 2.4% in mAP50 and mAP50-95, respectively, compared to the baseline model, while reducing the number of parameters by 65.3%. Its generalization capability for UAV object detection is further validated on the UAVDT and UAVVaste datasets. The proposed model is deployed on a typical airborne platform, Jetson Orin Nano, providing an effective solution for real-time object detection scenarios in actual UAVs.

## 1. Introduction

The rapid advancement and widespread deployment of Unmanned Aerial Vehicle (UAV) technology has positioned these platforms as critical infrastructure components across numerous application sectors, including intelligent urban infrastructure management [1,2], mineral and resource exploration [3], precision agricultural monitoring [4,5], disaster response and assessment [6], and perimeter security operations [7]. UAV-mounted imaging systems acquire substantial volumes of visual data from elevated vantage points, providing a crucial source of information for decision-making in these applications.

The task of real-time object detection in UAV aerial imagery presents multifaceted challenges that distinguish it from conventional object detection scenarios. Variations in flight altitude, viewing angle, and sensor focal length introduce substantial intra-class scale heterogeneity across acquired imagery, creating complex multi-scale object distributions that demand more efficient processing [2,3]. Targets in UAV aerial imagery are often extremely small [4]. In this study, small targets in UAV aerial imagery are defined based on their pixel area. Specifically, a target is considered small if its bounding box occupies fewer than 32 × 32 pixels. Small pixel areas lead to severe information sparsity, where discriminative features are highly compressed and prone to degradation during convolutional and downsampling operations. Additionally, unstructured backgrounds such as vegetation, urban areas, and water bodies introduce complex textures, illumination changes, and distractors, contributing to high false-positive and false-negative rates [6]. Furthermore, UAV platforms are subject to stringent hardware constraints—including limited computation, memory, and power—which necessitate highly efficient algorithms capable of balancing real-time performance with detection accuracy under embedded processing limitations.

Although traditional object detection architectures (including R-CNN [8], Faster R-CNN [9], SSD [10], and YOLO series [11]) have demonstrated robust performance on general scene benchmark datasets, their structures are mainly optimized for feature extraction of medium and large objects, and have limited representation capabilities for multi-scale and small object detection. The deep networks of general architectures bring huge memory burdens and computational requirements, making them difficult to deploy on resource-limited UAV platforms.

In recent years, a variety of real-time UAV detection architectures have been proposed. Weng et al. [12] introduced a multi-scale dilated attention mechanism and a GhostConv lightweight module to reduce algorithmic complexity while simultaneously enhancing multi-scale object feature extraction capabilities; Liu et al. [13] employed a gated single-head attention and large convolution kernel mechanism to achieve efficient extraction of local detail features; and Luo et al. [14] enhanced the model’s small object feature extraction capabilities while preserving computational efficiency by integrating multi-scale attention mechanisms and Shuffle block algorithms into the backbone network architecture. Although these methods have made some progress in improving the accuracy of real-time UAV target detection and reducing model complexity, there is still room for improvement in balancing detection accuracy and computational efficiency. Furthermore, these approaches fail to provide scalable model variants of varying computational complexity for practical deployment on UAV platforms with diverse computational resource constraints.

**Figure 1:**
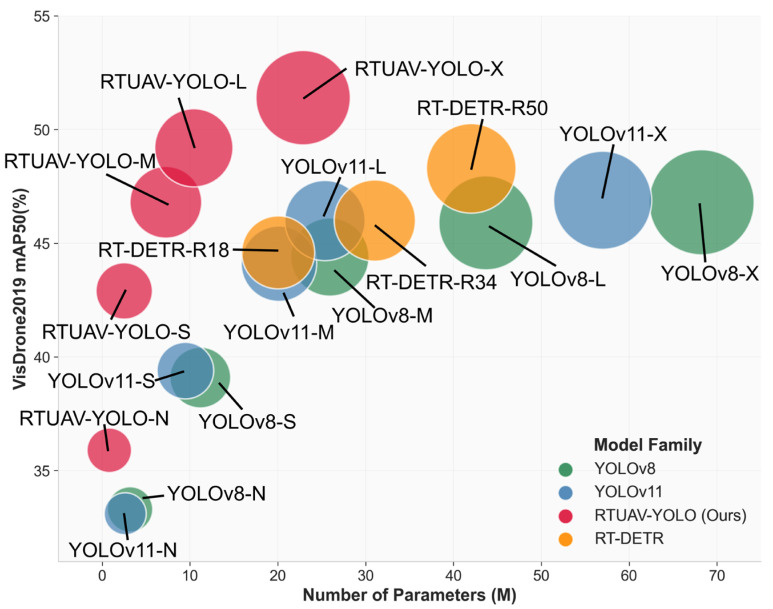
Comparison of RTUAV-YOLO with other models on the VisDrone2019 dataset. The circle radius represents GFlops.

To address these limitations, we propose a family of real-time UAV detection models, termed RTUAV-YOLO, based on YOLOv11 architecture. This model further improves detection accuracy and computational efficiency, and constructs models of varying scales to accommodate the practical deployment requirements of various UAV platform hardware. The principal contributions of this work can be summarized as follows:

(1) To address the loss of contextual information for small objects caused by multiple downsampling in YOLOv11, we introduced a Progressive Dilated Separable Convolution Module (PDSCM) in the P4–P5 phase of the backbone network. This module reduces feature sampling and uses expanding depth-wise separable convolutions to detect objects at different scales. It establishes contextual spatial relationships between these scales, enhancing the representation of small objects at multiple scales. A P2 detection head is introduced to the head, allowing the model to focus more on detailed features, thereby improving its sensitivity to small objects.
(2) To address the issues of unbalanced features and decreased information content in small-scale object processing in traditional architectures, we designed a lightweight Multi-Scale Feature Adaptive Modulation module (MSFAM) and replaced the C3K2 module in the backbone network. This significantly enhanced the feature extraction capability of small objects through an adaptive weight generation mechanism and dual-channel heterogeneous feature aggregation. We also introduced a Lightweight DownSampling Module (LDSM) to replace the convolutional modules in the backbone and Neck, achieving efficient downsampling of feature maps while reducing computational complexity and preserving key small-object features.
(3) To rectify the insensitivity of conventional Intersection over Union (IoU) metrics to-ward small objects, we develop a Minimum Point Distance Wise IoU (MPDWIoU) loss function. This loss function integrates minimum point distance metrics, dynamic anchor focusing strategies, and auxiliary bounding box supervision to specifically mitigate gradient imbalance and low precision in small-target localization while enhancing regression robustness in cluttered environments.
(4) Comprehensive experiments on the VisDrone2019 dataset confirms that RTUAV-YOLO achieves an effective balance between computational efficiency and detection performance. RTUAV-YOLO achieves an average improvement of 3.4% and 2.4% in mAP50 and mAP50-95, respectively, compared to the baseline model YOLOv11, while reducing the number of parameters by 65.3%. Comprehensive comparison results across different model series are presented in Figure 1. Furthermore, cross-dataset evaluations on the UAVDT and UAVVaste datasets confirm RTUAV-YOLO’s generalization and robustness across diverse UAV aerial imagery conditions. The complete implementation code is publicly available at https://gitee.com/zhangruizhi0110/RTUAV-YOLO (accessed on 20 October 2025).

## 2. Related Works

### 2.1. General Object Detection Models and Limitations

Contemporary deep learning-based object detection methodologies are categorized into two principal paradigms: two-stage and single-stage architectures. Two-stage frameworks encompass R-CNN [8], SPPNet [15], Fast R-CNN [16], and Faster R-CNN [9]. These frameworks demonstrate superior accuracy and robustness when processing complex visual scenes; however, they exhibit substantial computational demands and prolonged inference latency. Single-stage architectures formulate object detection as a unified regression task, directly predicting bounding box coordinates and class probabilities from input imagery without intermediate region proposal generation. This paradigm substantially enhances inference velocity while concurrently reducing computational complexity. The YOLO family of architectures [11] represents the most prominent exemplar of this paradigm. YOLO architectures have achieved widespread adoption in practical applications due to their speed–accuracy trade-off characteristics. The most recent iteration, YOLOv11 [17], enhances feature extraction and processing pipelines through the integration of novel architectural components including C3k2 and C2PSA modules, thereby achieving improved detection accuracy while simultaneously reducing parametric complexity and computational overhead, ultimately demonstrating superior performance on standard benchmarks such as COCO [18].

Nevertheless, direct application of these general architectures to UAV aerial imagery reveals several critical limitations: (1) Multi-scale and small objects detection deficiencies: Successive downsampling operations in deep networks result in substantial information loss for diminutive objects and inadequate exploitation of fine-grained shallow features. (2) Background interference susceptibility: Conventional feature extraction mechanisms and detection head architectures exhibit limited robustness against the heterogeneous backgrounds characteristic of aerial imagery. (3) Computational resource incompatibility: High-precision deep architectures impose substantial computational burdens that exceed the processing capabilities of resource-constrained UAV platforms, thereby precluding actual deployment.

While general object detection frameworks establish foundational detection paradigms, their architectural designs and optimization objectives are difficult to adapt to the inherent complex characteristics of UAV aerial images, necessitating domain-specific architectural innovations.

### 2.2. Object Detection Model for UAV Aerial Images

To address the shortcomings of general target detection models, researchers has developed numerous object detection architectures specifically tailored for UAV aerial imagery. Numerous approaches focus on enhancing multi-scale feature extraction capabilities to address small-target detection challenges. Li et al. [19] achieved multi-scale feature fusion by introducing the receptive field convolution block attention module and the balanced spatial semantic information fusion pyramid network, effectively balancing spatial and semantic information; Zhou et al. [20] introduced the non-semantic sparse attention (NSSA) mechanism and the bidirectional multi-branch auxiliary feature pyramid network to achieve significant improvement in small object detection performance; Guo et al. [21] presented AugFPN to mitigate the semantic-detail information disparity inherent in feature representations, employing single-pass supervision during feature fusion to reduce this informational discrepancy. While these approaches substantially enhance small-target recall performance, the incorporation of sophisticated architectural components frequently results in considerable computational overhead.

To mitigate false negatives and false positives arising from background complexity, Cheng et al. [22] employed dual attention mechanisms for pre-fusion feature enhancement, enabling selective focus on discriminative target characteristics; Zhang et al. [23] introduced a feature enhancement module leveraging spatial and channel attention mechanisms to augment feature representations, thereby improving target region localization; Li et al. [24] incorporated large-kernel convolutions coupled with dual-channel attention to enhance complex small-target feature extraction while effectively suppressing background interference. These attention-based methodologies substantially improve model robustness against environmental noise and background clutter.

To ensure that the designed UAV real-time target detection model meets the actual deployment requirements, model lightweight technology must be adopted. Model lightweight technology typically encompass two primary paradigms. The first approach involves structural pruning [25,26,27,28], which systematically eliminates redundant parameters below predefined thresholds through algorithmic filtering mechanisms. Pruning techniques offer universal applicability across diverse architectural frameworks for parameter reduction. The alternative paradigm employs lightweight modular design for structural optimization. This approach centers on developing computationally efficient architectural innovations. Prominent examples include MobileNet [29], ShuffleNet [30], and GhostNet [31] leverage depth-wise separable convolutions (DWConv) and grouped convolutions for spatial feature extraction. DWConv architectures achieve substantial reductions in both parametric complexity and floating-point operations (FLOPs). Architectural optimization has emerged as the predominant strategy in UAV real-time object detection model lightweight owing to its superior potential for achieving optimal speed–accuracy trade-offs.

Despite advances in UAV-specific detection frameworks, approaches that enhance small-target detection or background robustness typically incur significant computational costs, whereas lightweight variants exhibit suboptimal detection performance [32,33]. Consequently, there exists a critical need for innovative lightweight architectures specifically engineered for UAV aerial imagery that can achieve state-of-the-art detection performance within stringent computational constraints.

### 2.3. Bounding Box Regression Loss Function

Bounding box regression accuracy constitutes a critical determinant of detection performance, particularly for small objects exhibiting challenging localization characteristics. Contemporary IoU-based loss functions including GIoU [34], DIoU [35], and CIoU [36] enhance convergence properties and localization precision through the incorporation of auxiliary geometric information such as centroid distances and aspect ratio constraints. The WIoU family [37] (encompassing variants v1, v2, and v3) employs adaptive weighting mechanisms to enhance regression robustness. Focal-IoU [38] identifies the insufficient gradient contribution of conventional IoU loss for small targets and addresses this limitation through adaptive loss weighting strategies during small-target detection.

Despite their individual merits, these loss formulations exhibit limitations when addressing the unique challenges inherent in UAV-based small-target detection [39], including substantial IoU sensitivity to minute positional perturbations and gradient imbalance induced by extensive background interference proximate to target boundaries. Specifically, existing works demonstrates insufficient investigation into precise minimum point distance modeling and adaptive focusing strategies tailored for small-target instances. This gap motivated our development of the MPDWIoU loss function, which integrates minimum point distance metrics, dynamic attention mechanisms, and auxiliary supervision strategies. This formulation specifically targets enhanced small-target localization optimization while mitigating the inherent deficiencies of conventional approaches in UAV aerial surveillance contexts.

## 3. Methods

### 3.1. RTUAV-YOLO Overall Framework

**Figure 2:**
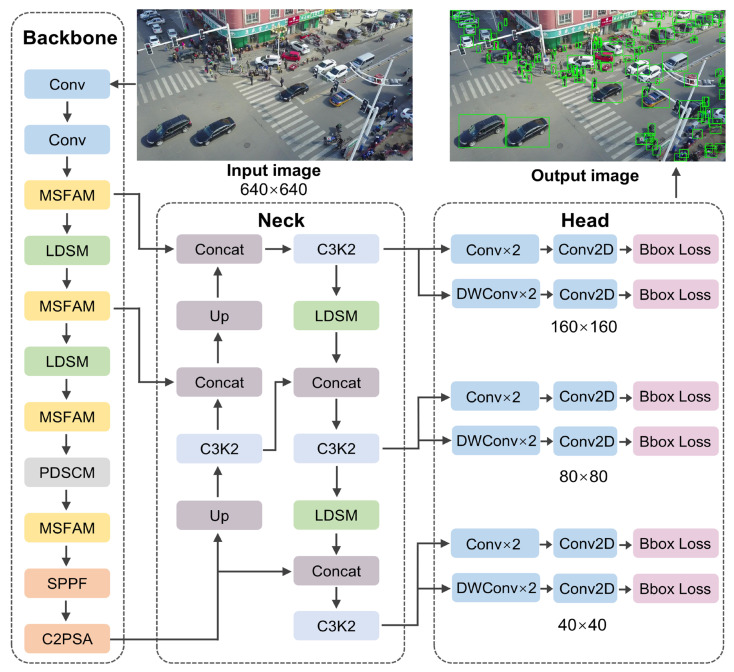
Overall structure of the RTUAV-YOLO.

Figure 2 illustrates the RTUAV-YOLO architecture presented in this work, which incorporates systematic enhancements to the YOLOv11 baseline framework. Initially, we replace the traditional convolutional module with the PDSCM in the P4 stage of the backbone network, eliminating the one-time downsampling of feature maps. The PDSCM promotes efficient aggregation of multi-scale receptive fields by integrating depth-wise separable convolutions with gradually increasing dilation rates, fully utilizing contextual information. We integrate the MSFAM as a replacement for the C3K2 components within the backbone architecture. The MSFAM substantially augments the model’s representational capacity and multi-scale target adaptability via three fundamental mechanisms: dynamic weight synthesis, multi-scale feature extraction, and adaptive feature modulation. Dynamic weight synthesis generates dynamic weight coefficients through adaptive average pooling and convolutional layers, dynamically allocating attention resources based on target scale and complexity. Multi-scale feature extraction utilizes a dual-branch parallel architecture to simultaneously extract and complement features at different scales. Adaptive feature modulation adaptively enhances useful features and suppresses redundant information based on input content, significantly improving the accuracy and robustness of small-target detection. The LDSM is deployed across both backbone and neck components. This module reduces parametric complexity and computational overhead via grouped and pointwise convolutions, simultaneously accomplishing spatial downsampling, feature aggregation, and channel-wise transformations. We incorporate the MPDWIoU loss function, which integrates minimum point distance metrics, dynamic attention weighting, and auxiliary supervisory mechanisms. These mechanisms specifically target gradient imbalance challenges in small-target localization while enhancing bounding box regression robustness against complex background interference. These lightweight modules, along with the introduction of IoU specifically for small object detection, enable RTUAV-YOLO to achieve excellent accuracy while reducing computational complexity. This significantly improves the model’s detection performance and generalization capabilities for UAV aerial imagery detection. The following sections will fully describe the architectural design and operating principles of each module.

### 3.2. Multi-Scale Feature Adaptive Modulation Module

In object detection applications, feature representational quality constitutes a fundamental determinant of detection efficacy. Conventional convolutional architectures encounter several critical limitations when addressing multi-scale object detection: (1) Limited content adaptivity: Static weight parameters fail to dynamically modulate feature significance based on contextual image characteristics. (2) Inadequate scale accommodation: Multi-scale objects necessitate diverse receptive field configurations and scale-specific feature processing mechanisms. (3) Suboptimal feature integration: Conventional concatenation or summation operations inadequately exploit the complementary nature of multi-scale feature representations. To mitigate these limitations, we introduce the MSFAM, grounded in adaptive attention theory and multi-scale feature integration principles. This module facilitates adaptive feature importance calibration through input-dependent dynamic weight learning while capturing multi-scale feature information via parallel multi-branch architectures. The MSFAM serves as a direct replacement for C3K2 modules within the YOLOv11 backbone, implementing computationally efficient feature extraction and fusion through reasonable architectural design.

Compared to the baseline YOLO and various YOLO-based UAV object detection models, these variants use fixed convolution kernels for multi-scale feature extraction, and their network designs at different depths do not optimize for semantic and positional information. Furthermore, their feature extraction modules do not dynamically prioritize features based on object scale. To address these issues, MSFAM utilizes a dynamic weight generator to enhance the relevance of features for small objects, a dual-branch feature extractor for efficient extraction and fusion of multi-scale information, and adaptive feature fusion for the effective fusion of semantic and positional information. This enables RTUAV-YOLO to better capture fine-grained details and contextual information, significantly improving the accuracy of small object detection in UAV imagery.

**Figure 3:**
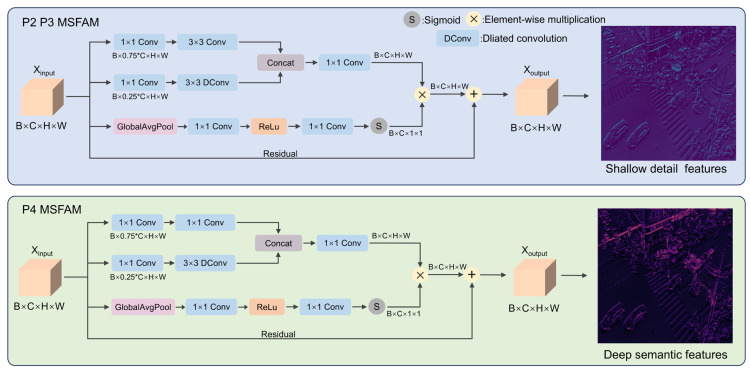
The specific architecture of MSFAM.

The MSFAM employs a three-stage cascaded architecture comprising three fundamental components: the Dynamic Weight Generator (DWG), Dual-Branch Feature Extractor (DBFE), and Adaptive Feature Fusion (AFF) mechanism. Given an input feature tensor $X\in R^{B\times C\times H\times W}$, the forward inference process of MSFAM is formulated as:

(1) Y=MSFAM(X)=AFF(DBFE(X),DWG(X))+X

where *B*, *C*, *H*, and *W* represent the batch size, number of channels, height, and width, respectively. Figure 3 shows the MSFAM used at different network depths.

#### 3.2.1. Dynamic Weight Generator

The Dynamic Weight Generator (DWG) leverages global average pooling coupled with a two-layer multilayer perceptron (MLP) to synthesize adaptive modulation weights from global feature statistics, enabling dynamic feature importance calibration. The DWG initially aggregates global statistical representations from input features via global average pooling:

(2) $$g=\frac{1}{HW}\sum_{i=1}^{H}\sum_{j=1}^{W}X_{:,:,i,j}$$

Subsequently, the two-layer MLP executes nonlinear transformations to accomplish dimensionality reduction and weight synthesis:

(3) $$h=\mathrm{ReLU}(W_{1}\cdot g+b_{1})$$

(4) $$w=\sigma (W_{2}\cdot h+b_{2})$$

where $W_{1}\in R^{C/r\times C}$, $W_{2}\in R^{C\times C/r}$, and r denotes the dimensionality reduction ratio. The reduction ratio r is adaptively configured based on network hierarchy, embodying the principle that shallow layers necessitate enriched feature representations while deeper layers prioritize computational efficiency.

To ensure weight stability and optimization efficacy, we employ L2 normalization:

(5) $$w_{norm}=\frac{w}{||w|{|}_{2}+\epsilon }$$

where ϵ represents a numerical stability constant to prevent division by zero. L2 normalization projects the weight vector onto the unit hypersphere, ensuring ${\left|w_{norm}|\right|}_{2}=1$, thus avoiding gradient explosion and providing a stable training process.

The adaptive weight modulation mechanism enables content-aware feature weight adjustment while generating channel-specific modulation coefficients, thereby amplifying responses in feature maps corresponding to small-target regions.

#### 3.2.2. Dual-Branch Feature Extractor

The dual-branch feature extractor employs a 3:1 channel allocation strategy coupled with an asymmetric dual-branch parallel architecture to facilitate efficient extraction and fusion of multi-scale information via distinct feature processing pathways. The specific implementation of the branch is differentiated according to the depth of the network. Shallow features exhibit high spatial resolution and rich detail information albeit with limited semantic content, whereas deep features demonstrate elevated semantic levels and abundant contextual information despite reduced spatial resolution. Within the shallow network layers P2 and P3, we implement a sophisticated feature extraction strategy to exploit the inherent richness of shallow features. Branch 1 employs standard 3 × 3 convolution to capture local detail features, while Branch 2 utilizes dilated convolution with a dilation rate of 2 to expand the receptive field and acquire broader contextual information. This architecture fully exploits the high-resolution characteristics inherent to shallow features. Its forward propagation process is as follows:

(6) $$F_{1}={\mathrm{Conv}}_{3\times 3}({\mathrm{Conv}}_{1\times 1}(X))$$

(7) $$F_{2}={\mathrm{DilatedConv}}_{3\times 3,d=2}({\mathrm{Conv}}_{1\times 1}(X))$$

Within the deep network layer P4, we adopt a streamlined feature extraction strategy to satisfy both semantic richness and computational efficiency requirements. Branch 1 employs two consecutive 1 × 1 convolutions for feature transformation while reducing computational complexity, whereas Branch 2 preserves the dilated convolution architecture to maintain contextual awareness. This architecture effectively balances semantic expressiveness with computational efficiency in deep network layers. Its forward propagation process is as follows:

(8) $$F_{1}={\mathrm{Conv}}_{1\times 1}({\mathrm{Conv}}_{1\times 1}(X))$$

(9) $$F_{2}={\mathrm{DilatedConv}}_{3\times 3,d=2}({\mathrm{Conv}}_{1\times 1}(X))$$

#### 3.2.3. Adaptive Feature Fusion

The adaptive feature fusion module seamlessly integrates information from the primary feature branch and the contextual branch. The module adaptively modulates feature importance according to target scale during small object feature extraction, dynamically amplifying the weights of small object-related features while maintaining stable performance enhancements via residual connections. This fusion mechanism not only enhances feature representation quality but also preserves computational efficiency and training stability. Initially, the output features from both branches are consolidated via channel concatenation:

(10) $$F_{\mathrm{concat}}=\mathrm{Concat}([F_{1},F_{2}],\dim=1)$$

Subsequently, a 1 × 1 convolution operation is applied to restore the original channel dimensionality:

(11) $$F_{\mathrm{fused}}={\mathrm{Conv}}_{1\times 1}(F_{\mathrm{concat}})$$

Incorporating the dynamic weights generated by the DWG, the final feature modulation process becomes:

(12) $$Y=w_{\mathrm{norm}}⊙F_{\mathrm{fused}}+X$$

where ⊙ denotes element-wise multiplication, and the residual connection guarantees gradient flow stability and comprehensive information preservation.

### 3.3. Progressive Dilated Separable Convolution Module

**Figure 4:**
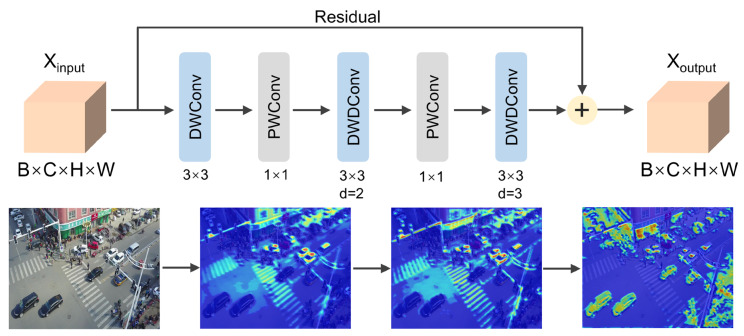
The specific architecture of PDSCM.

Small objects in UAV aerial imagery are frequently obscured by complex background interference, yielding constrained informative content. To comprehensively exploit available feature information, we implement the PDSCM to detect multi-scale objects and establish inter-scale spatial relationships, thereby augmenting contextual and small object feature representations. This module accomplishes multi-scale feature extraction and progressive receptive field expansion via a cascade of depth-wise separable convolutions with incrementally increasing dilation rates. This module is integrated within the P4 stage of the backbone architecture. The comprehensive architecture of the PDSCM and corresponding layer-wise feature extraction heatmaps are illustrated in Figure 4.

The PDSCM employs a progressive dilation rate strategy to accomplish incremental receptive field expansion across five convolutional layers. For dilated convolution operations, the effective receptive field is computed as:

(13) $$RF_{effective}=(k-1)\times d+1$$

Considering the concatenation of convolutional layers, the cumulative receptive field becomes:

(14) $$RF_{cumulative}=\sum_{i=1}^{n}RF_{i}-(n-1)$$

The resulting cumulative receptive field of the PDSCM yields $RF_{total}=3+5+7-2=13$.

The PDSCM leverages progressive dilation to process multi-scale information hierarchically. For local detail extraction, dilated convolutions with unit dilation rate capture fine-grained local features. For intermediate-scale contextual analysis, dilated convolutions with dilation rate 2 extract mid-level contextual information. For global contextual modeling, dilated convolutions with dilation rate 3 acquire large-scale global information. The final output incorporates residual connections, preserving information flow while enhancing training stability. Through this meticulously engineered architecture, the PDSCM achieves optimal balance between computational efficiency and feature representational capacity.

In contrast to conventional YOLO variants, which rely on standard convolution operations with fixed receptive fields, the PDSCM model progressively increases the dilation rate, thereby facilitating enhanced multi-scale context extraction. This approach proves especially advantageous for detecting small objects in complex UAV environments, where small targets often require larger receptive fields to be distinguished from cluttered backgrounds. By more effectively expanding the receptive field, PDSCM significantly improves small-object localization and enhances overall detection accuracy.

### 3.4. Lightweight DownSampling Module

The LSDM constitutes a purpose-built efficient downsampling component. This module executes the essential functions of spatial resolution reduction and channel dimensionality modulation within the RTUAV-YOLO architecture. It accomplishes lightweight feature downsampling via a meticulously engineered dual-stage convolutional framework.

**Figure 5:**
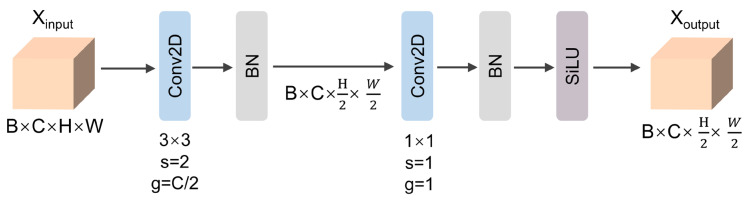
The specific architecture of LDSM.

The initial stage concentrates on spatial downsampling, whereas the subsequent stage addresses channel dimensionality modulation. This approach circumvents complex channel transformations during downsampling operations and enhances gradient backpropagation stability. Grouped convolution preserves feature locality, whereas pointwise convolution facilitates efficient inter-channel information exchange. Conventional convolution modules typically employ a “convolution + batch normalization + activation” paradigm, performing direct downsampling with stride 2. This nonlinear processing can distort or alias detailed features prior to downsampling, rendering it inappropriate for subtle small object textures. Conversely, the LDSM initially executes linear grouped convolution for downsampling, effectively implementing a “sample-first, nonlinearity-second” strategy that aligns more closely with signal processing principles and superior detail preservation. Subsequently, it executes channel fusion and nonlinear transformation via 1×1 pointwise convolution. This lightweight downsampling module decomposes complex operations into elementary, independent sub-operations, substantially reducing parametric complexity while maintaining feature representational fidelity. It proves particularly advantageous for applications demanding high-performance small object detection under computational resource constraints. The comprehensive architecture of the LSDM is illustrated in Figure 5. Unlike conventional YOLO variants that typically rely on standard convolutional layers for downsampling, LDSM minimizes distortions during downsampling by adopting a dual-stage convolutional framework, ensuring that small-object textures remain intact. This design enables RTUAV-YOLO to achieve more precise detection of small targets in UAV imagery, where maintaining fine spatial details is essential for accurate detection in complex and cluttered environments.

### 3.5. Minimum Point Distance Wise IoU

Intersection over Union (IoU) constitutes a fundamental metric for object detection evaluation and bounding box regression loss computation. However, conventional IoU variants exhibit constrained sensitivity to spatial misalignment in small object scenarios, yielding suboptimal detection performance for objects with minimal pixel coverage.

Conventional IoU and its variants exhibit the following critical limitations when processing small objects: (1) gradient vanishing: when predicted and ground-truth boxes lack intersection, IoU equals zero, yielding vanishing gradients; (2) inadequate small object sensitivity: minor pixel displacement substantially affects small objects, yet IoU variation remains negligible; (3) scale dependency: identical absolute displacement disproportionately impacts objects across varying scales. To address these limitations, we introduce MPDWIoU, a novel metric specifically engineered to address small object detection challenges. It integrates multi-dimensional geometric relationships, an adaptive focusing mechanism, and auxiliary IoU computation to deliver efficient localization guidance for small objects while preserving robustness across diverse object scales.

Assume that the two bounding boxes are:

(15) $$B_{1}=(x_{1}^{1},y_{1}^{1},x_{2}^{1},y_{2}^{1})$$

(16) $$B_{2}=(x_{1}^{2},y_{1}^{2},x_{2}^{2},y_{2}^{2})$$

where $\left(x_{1},y_{1}\right)$ and $\left(x_{2},y_{2}\right)$ represent the coordinates of the upper left corner and the lower right corner, respectively.

The width and height are defined as follows:

(17) $$w_{1}=x_{2}^{1}-x_{1}^{1},h_{1}=y_{2}^{1}-y_{1}^{1}$$

(18) $$w_{2}=x_{2}^{2}-x_{1}^{2},h_{2}=y_{2}^{2}-y_{1}^{2}$$

MPDWIoU establishes direct geometric constraints through the minimum point distance (MPD) mechanism. The fundamental principle involves computing the minimum Euclidean distance among all corner point pairs between predicted and ground-truth boxes. This distance directly quantifies the closest spatial proximity between predicted and ground-truth boxes, furnishing pixel-level positional constraints for model optimization. Specifically, the MPD mechanism extracts the coordinates of the four corner vertices from both predicted and ground-truth boxes, computes the Euclidean distances between all corner pairs to identify the minimum value, and subsequently employs the diagonal length of the minimum circumscribed rectangle encompassing both boxes for normalization to ensure scale invariance. This architectural design enables MPD to deliver more precise positional gradient signals compared to traditional IoU metrics, particularly when predicted boxes exhibit slight spatial offsets. While IoU variations remain minimal, MPD can furnish substantially clearer gradient signals to guide model optimization. The detailed computational procedure for MPD is delineated as follows.

Initially, the Euclidean distances between the four corresponding corner vertices are computed:

(19) $$d_{1}=\sqrt{(x_{1}^{1}-x_{1}^{2}{)}^{2}+(y_{1}^{1}-y_{1}^{2}{)}^{2}}$$

(20) $$d_{2}=\sqrt{(x_{2}^{1}-x_{2}^{2}{)}^{2}+(y_{1}^{1}-y_{1}^{2}{)}^{2}}$$

(21) $$d_{3}=\sqrt{(x_{1}^{1}-x_{1}^{2}{)}^{2}+(y_{2}^{1}-y_{2}^{2}{)}^{2}}$$

(22) $$d_{4}=\sqrt{(x_{2}^{1}-x_{2}^{2}{)}^{2}+(y_{2}^{1}-y_{2}^{2}{)}^{2}}$$

Subsequently, MPD is defined and the diagonal length of the minimum bounding convex hull is determined:

(23) $$\mathrm{MPD}=\min(d_{1},d_{2},d_{3},d_{4})$$

(24) $$c_{w}=\max(x_{2}^{1},x_{2}^{2})-\min(x_{1}^{1},x_{1}^{2})$$

(25) $$c_{h}=\max(y_{2}^{1},y_{2}^{2})-\min(y_{1}^{1},y_{1}^{2})$$

(26) $$c^{2}=c_{w}^{2}+c_{h}^{2}$$

The resultant MPD penalty term is formulated as:

(27) $$P_{\mathrm{mpd}}=\frac{\mathrm{MP}D^{2}}{c^{2}}$$

The aspect ratio penalty term is computed employing an adaptive weighting factor, leveraging the aspect ratio consistency component of CIoU:

(28) $$v=\frac{4}{\pi^{2}}{\left(\arctan\frac{w_{2}}{h_{2}}-\arctan\frac{w_{1}}{h_{1}}\right)}^{2}$$

(29) $$\alpha =\frac{v}{v-\mathrm{IoU}+1+\epsilon }$$

(30) $$P_{\mathrm{ar}}=\alpha \cdot v$$

To ensure enhanced attention allocation to small objects while preventing excessive penalization of high-quality samples, scale-adaptive weight computation is executed utilizing a small object threshold of 32 × 32 = 1024 pixels. This mechanism is subsequently integrated with quality-aware suppression to derive a dynamic focus weight. The corresponding computational formulation is expressed as follows:

(31) $$A_{1}=w_{1}\cdot h_{1},\;\;A_{2}=w_{2}\cdot h_{2}$$

(32) $$A_{\min}=\min(A_{1},A_{2})$$

(33) $$\beta =\frac{A_{\min}}{A_{\min}+1024}$$

(34) γ=2−2⋅IoU

(35) $$W_{f}=\beta \cdot \exp(-\gamma )$$

The Inner-IoU enhancement mechanism augments the model’s sensitivity to positional variations in small objects through the construction of a scaled auxiliary bounding box. Through computing the IoU between the predicted box and the scaled ground-truth box, followed by weighted fusion with the original IoU, the precision of small object detection is substantially enhanced. The corresponding mathematical expression is delineated as follows, where the scaling parameter s = 1.25:

(36) $${\hat{w}}_{1}=s\cdot w_{1},\;\;{\hat{h}}_{1}=s\cdot h_{1}$$

(37) $${\hat{w}}_{2}=s\cdot w_{2},\;\;{\hat{h}}_{2}=s\cdot h_{2}$$

(38) $$c_{1}^{x}=\frac{x_{1}^{1}+x_{2}^{1}}{2},c_{1}^{y}=\frac{y_{1}^{1}+y_{2}^{1}}{2}$$

(39) $$c_{2}^{x}=\frac{x_{1}^{2}+x_{2}^{2}}{2},c_{2}^{y}=\frac{y_{1}^{2}+y_{2}^{2}}{2}$$

(40) $$B_{1^{\prime }}=\left(c_{1}^{x}-\frac{{\hat{w}}_{1}}{2},c_{1}^{y}-\frac{{\hat{h}}_{1}}{2},c_{1}^{x}+\frac{{\hat{w}}_{1}}{2},c_{1}^{y}+\frac{{\hat{h}}_{1}}{2}\right)$$

(41) $$B_{2^{\prime }}=\left(c_{2}^{x}-\frac{{\hat{w}}_{2}}{2},c_{2}^{y}-\frac{{\hat{h}}_{2}}{2},c_{2}^{x}+\frac{{\hat{w}}_{2}}{2},c_{2}^{y}+\frac{{\hat{h}}_{2}}{2}\right)$$

(42) $$\mathrm{Io}U_{\mathrm{aux}}=\mathrm{IoU}(B_{1^{\prime }},B_{2^{\prime }})$$

Integrating all constituent elements, MPDWIoU is formulated as:

(43) $$\mathrm{MPDWIoU}=\mathrm{IoU}-P_{\mathrm{mpd}}-P_{\mathrm{ar}}+W_{f}\cdot (\mathrm{Io}U_{\mathrm{aux}}-\mathrm{IoU})$$

MPDWIoU addresses the fundamental gradient vanishing problem inherent in traditional IoU metrics under non-intersecting conditions via the minimum point distance mechanism. The framework incorporates dynamic focus weights to achieve adaptive attention allocation for small targets while augmenting boundary localization sensitivity through an integrated scaling mechanism. The method substantially enhances small-target detection accuracy and training stability while preserving computational efficiency, thereby furnishing a more precise and robust loss function solution for object detection in small-target-dense environments.

## 4. Experiments and Results

### 4.1. Datasets

**Figure 6:**
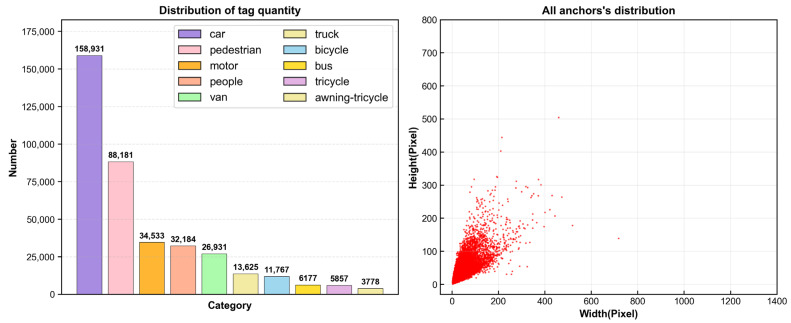
Object type and size distribution of the VisDrone2019 dataset.

To validate the efficacy of the proposed RTUAV-YOLO model, comprehensive comparative experiments were conducted utilizing the publicly available VisDrone2019 dataset [40], with model generalization capabilities further assessed on two additional datasets: UAVDT [41] and UAVVaste [42]. The VisDrone2019 dataset was developed by the AISKYEYE team within the Machine Learning and Data Mining Laboratory at Tianjin University. It is a large-scale UAV aerial image benchmark dataset collected under various low-altitude conditions using UAV. The dataset encompasses imagery acquired across varied real-world scenarios, diverse meteorological conditions, and multiple target scales. The dataset comprises ten distinct object categories: pedestrians, people, bicycles, cars, trucks, motorcycles, vans, tricycles, awning-tricycles, and buses. Specifically, the dataset contains 6471 training images, 548 validation images, and 3190 test images. Figure 6 illustrates the distribution of annotation categories and object size characteristics. The primary detection challenges inherent in this dataset manifest in several key aspects: (1) the substantial quantity of objects requiring detection, predominantly small-scale targets (defined as objects with bounding boxes smaller than 32 × 32 pixels); (2) significant inter-object occlusion and spatial overlap; (3) imbalanced class distribution. While these challenges substantially increase detection complexity, the dataset’s inherent richness and diversity establish a robust foundation for comprehensive model performance evaluation. The training and validation subsets of VisDrone2019 were employed for model training and performance assessment.

UAVDT constitutes a large-scale benchmark dataset specifically designed for unmanned aerial vehicle-based detection and tracking applications. This comprehensive dataset, comprising approximately 80,000 representative frames meticulously curated from 10 h of raw video footage, is purposefully constructed to address emerging challenges inherent in complex visual scenarios. The dataset incorporates comprehensive bounding box annotations alongside up to 14 attribute annotations (including meteorological conditions, flight altitude, camera perspective, vehicle classification, and occlusion characteristics), thereby supporting three fundamental computer vision tasks: object detection (DET), single object tracking (SOT), and multiple object tracking (MOT). The dataset encompasses annotations for approximately 2700 vehicles, totaling approximately 840,000 bounding boxes, thus establishing a crucial benchmark for aerial object detection and tracking research. This investigation utilizes the object detection subset of the dataset, which comprises 38,327 images with an average spatial resolution of 1080 × 540 pixels. The dataset encompasses three distinct object categories: passenger vehicles, buses, and trucks. The dataset comprises 23,258 training images and 15,069 evaluation images. UAVVaste constitutes a specialized dataset purposefully designed for aerial debris detection applications. This dataset contains 772 images and 3716 annotated landmarks depicting debris across diverse urban and natural environments, including streets, parks, and residential areas. For both datasets, the training were employed for model training, while the test were utilized for performance evaluation.

### 4.2. Implementation Details

All experiments were conducted on a high-performance computational server equipped with an Intel Core i7-14700K CPU and an NVIDIA GeForce RTX 4090 GPU. The software environment was built on Ubuntu 22.04, with Python 3.8, PyTorch 2.0, and CUDA 11.8.

Our model was trained from scratch for 300 epochs using a Stochastic Gradient Descent (SGD) optimizer. The batch size was set to 16, and the number of data loading workers was configured to 8. Input images were uniformly resized to 640 × 640 pixels to balance detection accuracy and computational load.

Key training hyperparameters were configured as follows: the optimizer momentum was set to 0.937 and the weight decay was 0.0005. The learning rate scheduling employed a linear warmup for the initial 3 epochs, followed by a cosine annealing strategy, starting with an initial learning rate of 0.01 and decaying to a final learning rate of 0.0001. An early stopping mechanism with a patience of 50 epochs was implemented to terminate the training if no improvement in validation metrics was observed, ensuring the selection of the best-performing model weights. For experimental consistency, all baseline models were trained under the identical hyperparameter configuration.

To enhance model robustness and prevent overfitting, we employed a comprehensive suite of data augmentation techniques. These included mosaic augmentation (enabled for the entire training duration except for the final 10 epochs), as well as random perspective shifts, hue, saturation, and value adjustments.

**Figure 7:**
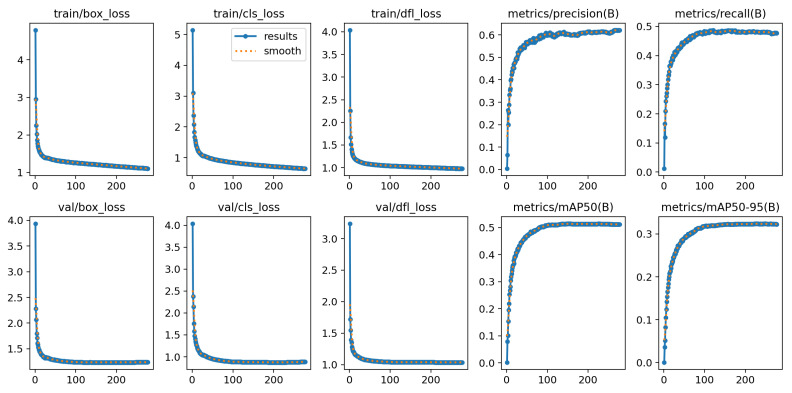
RTUAV-YOLO-X training results at VisDrone2019 dataset.

Taking the RTUAV-YOLO-X version as an example, Figure 7 shows its training results on the VisDrone2019 dataset. The maximum number of epochs configured for the entire training process was 300. However, due to the activation of the early stopping mechanism, training automatically terminated after 278 epochs. The total training time was 78,892.3 s, or approximately 21.92 h. This translates to an average training time of approximately 284 s per epoch. The model demonstrated stable convergence during training. The key performance metric, mAP50-95, showed a continuous upward trend, starting from 0.116 in the first epoch and peaking at approximately 0.324 in the 227th epoch. Thereafter, mAP50-95 began to stabilize with only minor fluctuations. Following the early stopping mechanism, validation set performance showed no improvement for 50 consecutive epochs, and training was terminated at epoch 278. This indicates that the model has reached its optimal convergence point, and further training may result in diminishing returns or potential overfitting. Analysis of the loss curves shows that the training process is stable and successful, without common problems such as gradient vanishing, and ultimately a detection model with strong generalization ability and excellent performance is constructed.

### 4.3. Evaluation Metrics

To comprehensively assess the efficacy of RTUAV-YOLO for small object detection tasks, this investigation employs precision (*P*), recall (*R*), mean average precision (mAP), parameter count (Params), and computational complexity as quantitative evaluation metrics.

#### 4.3.1. Precision

Precision quantifies the proportion of correctly identified objects relative to the total number of predicted detections, thereby characterizing the model’s classification accuracy. The mathematical formulation is expressed as:

(44) $$P=\frac{TP}{TP+FP}$$

where *TP* denotes the count of true positive instances (correctly classified positive samples), and *FP* represents the count of false positive instances (incorrectly classified negative samples as positive).

#### 4.3.2. Recall

Recall quantifies the proportion of correctly identified positive instances relative to the total number of actual positive samples, thereby indicating the model’s detection sensitivity and coverage capability. The mathematical expression is formulated as:

(45) $$R=\frac{TP}{TP+FN}$$

where *FN* is the number of samples predicted as negative but actually positive.

#### 4.3.3. Mean Average Precision

Average Precision (AP) quantifies the detection performance for individual object categories by computing the area under the precision-recall curve, thereby capturing the trade-off relationship between precision and recall metrics. Mean Average Precision (mAP) provides a comprehensive assessment of the model’s overall detection capability by statistically aggregating the average precision values across all target categories. The computational framework is expressed as:

(46) $$\mathrm{AP}=\int_{0}^{1}P\cdot \mathrm{RdR}$$

(47) $$mAP=\frac{1}{C}\sum_{c=1}^{C}AP_{c}$$

where C denotes the total number of object categories within the detection framework, and $AP_{c}\;$represents the average precision metric for the C category target. To provide a more comprehensive performance evaluation, this investigation employs two distinct mAP metrics: mAP50 quantifies the mean average precision at a fixed IoU threshold of 0.5, while mAP50-95 represents the averaged precision across multiple IoU thresholds ranging from 0.5 to 0.95 with incremental steps of 0.05.

#### 4.3.4. Model Parameter Scale

Model parameter count quantifies the total number of trainable parameters within the neural network architecture, serving as a fundamental metric for assessing model complexity, memory requirements, and computational overhead. Reduced parameter counts enable efficient model deployment in resource-limited computational environments, thereby enhancing practical applicability.

#### 4.3.5. Floating Point Operations

Computational complexity, quantified through floating-point operations per second, represents the aggregate number of arithmetic operations executed by the neural network architecture during a single forward propagation cycle, typically expressed in giga-FLOPs (GFLOPs). Reduced GFLOPs requirements correspond to enhanced computational efficiency during inference, thereby facilitating deployment in resource-constrained real-time detection scenarios, particularly within UAV platforms where computational resources are inherently limited.

### 4.4. Comparative Experiments

#### 4.4.1. Experimental Results on the VisDrone2019 Dataset

To rigorously assess the efficacy of the proposed methodology, comprehensive comparative evaluations were conducted across diverse state-of-the-art (SOTA) object detection architectures utilizing the VisDrone2019 dataset.

The quantitative comparison results with the baseline model, presented in Table 1, encompass performance metrics for the YOLOv8 [43] family, YOLOv11 [17] family, and the proposed RTUAV-YOLO series architectures.

With respect to mAP50 evaluation, RTUAV-YOLO-X demonstrated superior detection performance among all evaluated architectures, attaining 51.4% accuracy. This constitutes a substantial improvement of 4.5 percentage points (9.6% relative enhancement) over the baseline YOLOv11-X performance of 46.9%. Under the more rigorous mAP50-95 evaluation protocol, RTUAV-YOLO-X maintained exceptional performance, achieving 32.4%, corresponding to a significant improvement of 3.4 per-centage points (11.7% relative gain) over YOLOv11-X’s baseline of 29.0%. Notably, the RTUAV-YOLO architecture family exhibited consistent performance superiority across all model scales.

The RTUAV-YOLO architecture series exhibits an optimal precision-recall equilibrium. Specifically, RTUAV-YOLO-X attains precision and recall values of 61.3% and 48.1%, respectively, corresponding to improvements of 3.6 and 3.3 percentage points over YOLOv11-X’s baseline performance (57.7% precision, 44.8% recall). These results substantiate that the proposed methodology not only mitigates false positive occurrences but also significantly reduces false negative instances, thereby enhancing overall detection robustness.

Comprehensive complexity analysis demonstrates that the RTUAV-YOLO architecture achieves superior detection performance while maintaining exceptional parameter efficiency and computational optimization. Regarding computational overhead, except for RTU-AV-YOLO-N, which exhibits marginally elevated FLOP requirements compared to YOLOv11-N, all remaining RTUAV-YOLO variants demonstrate reduced computational complexity relative to their corresponding baseline architecture. Specifically, the RTUAV-YOLO-X variant comprises 22.86 M parameters with a computational complexity of 168.1 G FLOPs. Relative to the corresponding YOLOv8-X and YOLOv11-X architectures, our proposed model shows a 66.5% and 59.9% reduction in parameters and a 35.0% and 14.2% reduction in FLOPs, while simultaneously achieving marked performance enhancements across all evaluation metrics.

**Figure 8:**
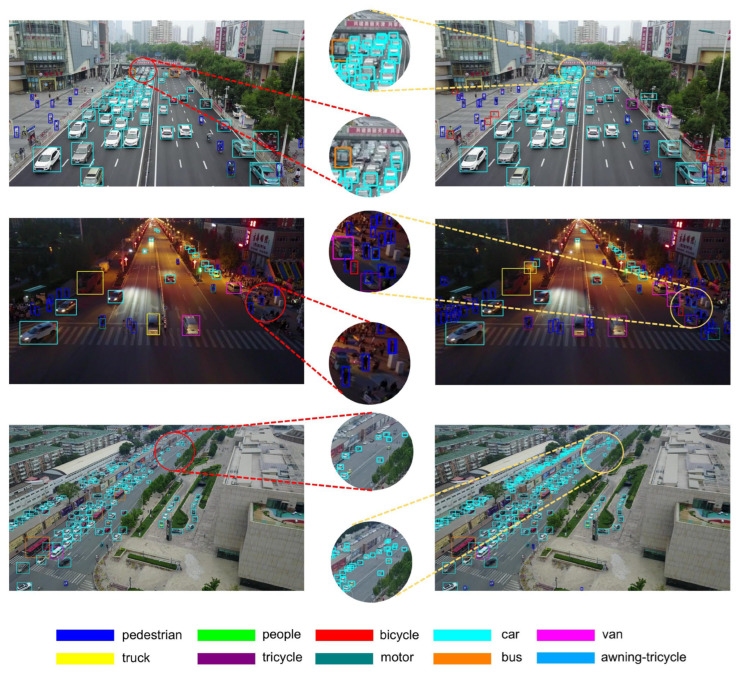
Visual comparison of detection results. The left side shows the results of YOLOv11-S, the right side shows the results of RTUAV-YOLO-S, and the center shows a detailed comparison of detection details.

To provide intuitive validation of the differential detection capabilities, representative images from diverse scenarios were selected for comprehensive visual analysis. The comparative results are presented in Figure 8, with the baseline YOLOv11-S model displayed on the left and the proposed RTUAV-YOLO-S architecture on the right. These visualizations demonstrate that our proposed method achieves satisfactory detection accuracy. Notably, the proposed approach successfully identifies numerous small objects frequently undetected by conventional detection algorithms, as evidenced in the second column visualization.

**Figure 9:**
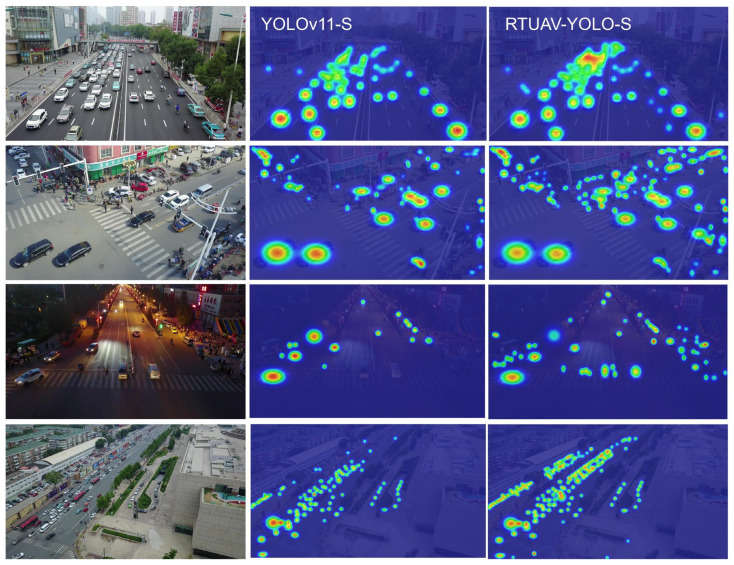
Visual heatmap analysis of YOLOv11-S and RTUAV-YOLO-S.

Figure 9 shows heatmaps of small objects in the VisDron2019 dataset. Relative to the baseline architecture, RTUAV-YOLO exhibits substantially enhanced capabilities for localizing densely clustered small objects. In our model’s heatmaps, small objects display higher heat values, indicating that our model is more effective in capturing the characteristics of these small objects. Furthermore, empirical observations reveal that RTUAV-YOLO exhibits enhanced attention to contextual information surrounding small targets, reflecting superior contextual feature utilization during the detection process.

To provide comprehensive performance validation, extensive comparative analysis was conducted on the VisDrone2019 dataset, benchmarking the RTUAV-YOLO model family against state-of-the-art aerial object detection architectures. These comparative models primarily include PS-YOLO [44], MSUD-YOLO [45], LGFF-YOLO [46], FBRT-YOLO [47], RT-DETR [48], CSFPR-RTDETR [49] and UAV-DETR [50] model. The comparison results are shown in Table 2. “-” indicates that the model was not proposed in the original literature.

Regarding detection accuracy, the RTUAV-YOLO architectural family demonstrates substantial performance enhancements across all model scales. RTUAV-YOLO-X attained superior performance among all evaluated architectures, achieving a mAP50 of 51.4%, representing a 3.0 percentage point improvement over FBRT-YOLO-X (48.4%). Under the more rigorous mAP50-95 evaluation criterion, RTUAV-YOLO-X maintained superiority over all competing architectures with a score of 32.4%. Notably, the lightweight RTUAV-YOLO-N variant outperforms comparable PS-YOLO-N and FBRT-YOLO-N architectures with a mAP50 of 35.9%, exemplifying exceptional performance in compact model configurations.

#### 4.4.2. Experimental Results on UAVDT and UAVVaste Datasets

To comprehensively assess the generalization performance of the proposed RTUAV-YOLO architecture for small object detection, extensive comparative evaluations were conducted across two distinct aerial imaging datasets: UAVDT and UAVVaste. These datasets exhibit substantial variations from VisDrone2019 regarding scene complexity, object scale distributions, and acquisition conditions, thereby providing rigorous validation of the model’s cross-domain adaptability and robustness. To maintain experimental consistency and ensure comparative validity, identical training protocols and hyper-parameter configurations were employed as established in the VisDrone2019 evaluation framework. Evaluation metrics included key performance indicators such as mAP50, mAP75, and mAP50-95. Across both datasets, the RTUAV-YOLO-X variant was employed for evaluation. Comparative performance results are presented in Table 3 and Table 4.

Evaluation on the UAVDT dataset demonstrates that RTUAV-YOLO-X attains superior performance across all assessment criteria. Specifically, RTUAV-YOLO-X attains 31.8% mAP50, representing a 0.7 percentage point enhancement over the second-best performing method, FBRT-YOLO (31.1%). Notably, under the more rigorous mAP75 criterion, which imposes stringent localization accuracy requirements, RTUAV-YOLO-X achieves 19.3%, substantially surpassing all comparative baselines and conclusively validating the proposed methodology’s efficacy. Under the comprehensive mAP50-95 evaluation framework, RTUAV-YOLO-X achieves 18.8%, representing a substantial 3.0 percentage point advancement over the YOLOv11-X baseline (15.8%), thereby establishing a marked performance superiority. Evaluation on the UAVVaste dataset provides additional validation of RTUAV-YOLO’s architectural superiority. RTUAV-YOLO-X attained 76.8% mAP50, demonstrating a 3.3 percentage point enhancement relative to RT-DETR-R50 (73.5%). This substantial performance gain substantiates the proposed methodology’s robustness across diverse aerial imaging scenarios. Under the comprehensive mAP50:95 assessment, RTUAV-YOLO-X achieved 38.2%, representing a remarkable 10.4% advancement over the YOLOv11-S baseline (27.8%). This substantial performance improvement fully demonstrates the technical advantages of the proposed method.

Comparative analysis across three distinct datasets reveals that RTUAV-YOLO exhibits exceptional cross-domain generalization capabilities. Primarily, RTUAV-YOLO maintains consistent performance superiority across all datasets, substantiating the architectural versatility of the proposed framework.

Furthermore, performance gains relative to baseline methods exhibit positive correlation with dataset complexity, thereby corroborating the methodology’s robustness in addressing challenging small object detection scenarios. Additionally, consistent performance across varying IoU thresholds demonstrates that RTUAV-YOLO not only achieves effective small object detection but also delivers enhanced localization precision, thereby establishing significant practical value for real-world aerial applications.

### 4.5. Ablation Experiments

To systematically validate the efficacy of individual architectural components within RTUAV-YOLO, comprehensive ablation studies were conducted on the VisDrone2019 dataset. These evaluations utilized YOLOv11-S as the baseline architecture and implemented a progressive module integration methodology. Initially, backbone network modifications were implemented, subsequently followed by the systematic integration of three novel modules: LDSM, MSFAM, and PDSCM. Subsequently, the bounding box regression loss function was substituted with the MPDWIoU formulation. Excluding the ablated components, training protocols and hyperparameter configurations remained consistent throughout the evaluation. Performance assessment employed mAP50 and mAP50-95 metrics, with parameter count and FLOPs serving as computational efficiency indicators. To verify the statistical significance of the ablation experiment results, we conducted multiple independent experiments on different combinations of each module (each experiment was run 3 times). The data in Table 5 are the average values of multiple independent experiments.

Empirical findings from our ablation studies show that architectural modifications to the backbone network significantly improve the detection of small objects by eliminating one downsampling operation, which preserves high-resolution feature maps. This adjustment also reduces the parameter count while only marginally increasing computational complexity. When progressively integrating modules, the LDSM contributes to the improved extraction of small target features by reducing computational complexity, thereby improving detection accuracy while maintaining efficiency. The MSFAM module further enhances feature extraction by fusing multi-scale features and dynamically adjusting feature importance. This leads to significant improvements in the detection of small objects across different scales.

The PDSCM module expands the spatial contextual relationships between small objects at multiple scales, further enhancing the model’s multi-scale representation capability and improving overall detection performance. When substituting the conventional IoU loss with MPDWIoU, we observe substantial improvements in bounding box regression, particularly for high-IoU regimes, without introducing additional parameters.

By analyzing the combined effects of each module, we found that while the introduction of a single module can bring a certain degree of performance improvement, it is the synergy of the modules that truly enables RTUAV-YOLO to achieve optimal performance. The results in Table 5 clearly demonstrate the modularity and synergistic complementarity of the various components. The independent contributions of each module are combined to significantly improve detection accuracy while maintaining the model’s computational efficiency. Our approach achieves an optimal balance between performance and efficiency, making it well-suited for resource-constrained drone deployment scenarios. The consistency of these results, supported by statistical analysis, further validates the robustness and reliability of the proposed method across various configurations.

Furthermore, to further validate the effectiveness of the proposed MPDWIoU loss, we conducted a comparative experiment with several widely used IoU-based loss functions, including GIoU, DIoU, CIoU, and WIoUv3. All models were trained under identical settings and datasets (VisDrone2019) using the RTUAV-YOLO-S configuration. The experimental results presented in Table 6 demonstrate that MPDWIoU achieves the highest detection accuracy on small targets, improving mAP50 by 0.6% and mAP50-95 by 0.4% compared to the GIoU loss. This improvement verifies that the proposed MPDWIoU effectively enhances localization precision and gradient stability for small objects through the introduction of minimum point distance constraints and adaptive weighting mechanisms.

### 4.6. Edge Computing Platform Deployment

**Figure 10:**
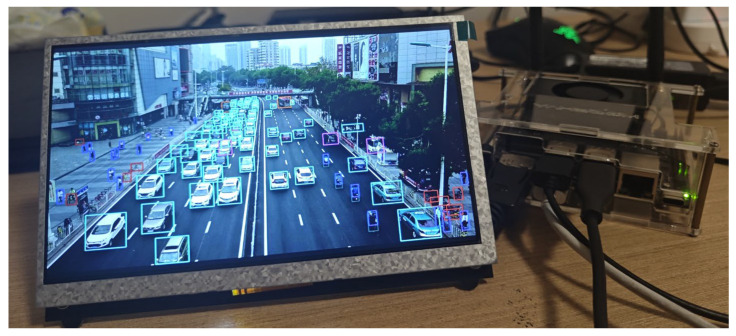
Actual deployment diagram of RTUAV-YOLO on Jetson Orin Nano.

To validate the deployment feasibility of the proposed RTUAV-YOLO architecture on edge computing platforms, comprehensive evaluation was conducted using the NVIDIA Jetson Orin Nano development board (NVIDIA, Santa Clara, CA, USA). Despite its compact form factor, the Jetson Orin Nano integrates 32 Tensor Cores and 1024 CUDA Cores, delivering substantial computational performance with optimized power efficiency. This configuration enables efficient computational processing for real-time object detection applications in UAV edge computing scenarios. The actual deployment and detection diagram of RTUAV-YOLO-S on NVIDIA Jetson Orin Nano is shown in Figure 10. The RTUAV-YOLO-S variant, when deployed on this edge computing platform, achieved a real-time inference rate of 37.8 FPS, satisfying real-time processing requirements while demonstrating an optimal balance between detection precision and computational efficiency.

To better evaluate the practical application of RTUAV-YOLO on a drone edge platform, we deployed several different real-time drone detection models on the device and quantitatively evaluated their detection accuracy and speed. The results are shown in Table 7. Compared with various advanced models, our proposed RTUAV-YOLO can better capture target features in complex scenes and achieve higher detection accuracy. Furthermore, its detection frame rate is better than that of most real-time drone models, meeting real-time detection requirements and being suitable for most practical drone application scenarios.

## 5. Discussion

### 5.1. Resource Suitability Under UAV Constraints

UAV onboard deployment is constrained by memory capacity, energy budget, and real-time requirements. To make these constraints explicit without relying on device-specific power probes, we use three hardware-agnostic proxies that are standard in efficient vision: parameter count (Params) to approximate the lower bound of model memory devoted to weights, floating-point operations (GFLOPs) to reflect per-image computational work and thus serve as an energy proxy under matched settings, and frames per second (FPS) to represent realized runtime on target hardware. We report these metrics for all comparison models and RTUAV-YOLO variants on a desktop GPU and a Jetson Orin Nano, and the results are shown in Table 1, Table 2 and Table 6.

In practical terms, fewer parameters indicate a smaller weight footprint and lighter data movement; lower GFLOPs indicate less arithmetic per image, which generally correlates with lower energy cost and latency; and higher FPS indicates stronger real-time feasibility under the same input resolution and batch size. Across our experiments, RTUAV-YOLO attains higher accuracy with markedly fewer parameters, lower or comparable GFLOPs, and higher FPS than comparable baselines on both platforms, indicating a favorable accuracy–efficiency balance for resource-constrained UAV scenarios.

We note that absolute power draw and peak activation memory depend on device configuration (e.g., precision, clocking, thermal limits) and deployment choices (e.g., batch size, resolution). The above proxies, together with on-device FPS, provide transparent and comparable evidence of suitability without over-claiming device-specific wattage. We plan to conduct rigorous memory and power analysis of RTUAV-YOLO deployed on actual drone hardware in subsequent research.

### 5.2. Resolution Choice and Small-Object Fidelity

Many remote-sensing scenes contain targets only a few pixels in size; when such imagery is resized to 640 × 640, fine-grained shape cues may be attenuated or lost. Our choice of a 640 × 640 input reflects the constraints of onboard UAV deployment—memory, energy, and strict real-time budgets—yet we explicitly consider the implications for small-object detection and through reasonable architectural design and appropriate data augmentation techniques, our proposed method mitigates the impact of this issue to a certain extent.

In addition to resource considerations, we adopt 640 × 640 to ensure apples-to-apples comparisons with prior art. The majority of recent YOLO-based detectors and widely used baselines for UAV/aerial benchmarks report results at this input size. Using the same resolution standardizes pre-processing, keeps mAP and runtime comparisons fair across methods, and aligns with community practice. Where relevant, we also provide results at a higher input size to quantify the accuracy–efficiency trade-off.

We quantify the fidelity–efficiency balance by comparing 640 × 640 versus 1280 × 1280 inputs on RTUAV-YOLO-S. At 640 × 640, training time per epoch was 126 s, throughput 162 FPS, with mAP50 was 42.9% and mAP50–95 was 25.9%. At 1280 × 1280, training time increased to 576 s, throughput decreased to 47 FPS, and accuracy rose modestly to mAP50 was 44.3% and mAP50-95 was 26.8%. Thus, larger inputs provide small gains in accuracy but impose substantial computational and latency costs that are incompatible with many real-time UAV missions.

Adopting 640 × 640 is a pragmatic operating point preserves essential target cues for most scenarios, matches the prevailing evaluation protocol for comparable methods, and meets real-time and resource constraints on typical UAV hardware. For missions where tiny-object fidelity dominates over latency or energy, higher input resolutions, tiling/windowing strategies, or adaptive-resolution policies are viable alternatives and constitute promising directions for future work.

### 5.3. Cross-Domain Generalization Capability Verification

To further evaluate the cross-domain generalization capability of the proposed RTUAV-YOLO architecture, we conducted additional experiments on the HIT-UAV [56] and AI-TOD [57] datasets, which are widely adopted benchmarks in UAV-based small object detection. Compared with the VisDrone2019, UAVDT, and UAVVaste datasets used in the main experiments, HIT-UAV and AI-TOD present markedly different imaging characteristics in terms of image resolution, viewing angle, background complexity, and detection targets. This enables a more comprehensive validation of the model’s robustness and adaptability to data distributions that differ significantly from the original training domain.

For consistency and fairness, we maintained identical training hyperparameters and evaluation metrics as described in Section 4.2. Table 8 and Table 9 report the quantitative detection results of RTUAV-YOLO and baseline YOLO series models on HIT-UAV and AI-TOD, respectively. The results show that RTUAV-YOLO maintains leading performance on the HIT-UAV and AI-TOD datasets. This demonstrates the strong generalization capabilities of our proposed model and its ability to effectively adapt to data from different distributions. In the future, we will pay more attention to the generalization ability verification of the model, which is crucial for building a general drone-view target detection model.

## 6. Conclusions

This study addresses the dual challenges of diminished small-target detection accuracy in UAV imagery and constrained computational resources on onboard platforms and present a comprehensive family of lightweight detection models, designated as RTUAV-YOLO. We designed three efficient lightweight modules: MSFAM, LDSM, and PDSCM. To address the loss of contextual information for small objects caused by multiple downsampling in YOLOv11, we used the PDSCM to reduce feature sampling from P4 to P5 and employed extended depth-wise separable convolutions to establish spatial contextual relationships between multiple scales, enhancing the representation of small objects at multiple scales. We introduced a P2 detection head within the detection head, enabling the model to focus more on detailed features, thereby improving sensitivity to small objects. To address the feature imbalance and information loss associated with traditional architectures when processing small objects, we replaced the C3K2 module in the backbone network with the MSFAM. This module significantly improves feature extraction for small objects through an adaptive weight generation mechanism and dual-channel heterogeneous feature aggregation. We also replaced the traditional convolutional modules in the backbone and neck with the LDSM, achieving efficient downsampling of feature maps while reducing computational complexity and preserving key small object features. To address the insensitivity of the traditional IoU metric to small objects, we designed the MPDWIoU loss function. This loss function integrates a minimum point distance metric, a dynamic anchor focus strategy, and auxiliary bounding box supervision to specifically alleviate gradient imbalance and low accuracy in small object localization, while enhancing regression robustness in cluttered environments.

Comprehensive experimental validation on the VisDrone2019 dataset confirms that RTUAV-YOLO achieves an effective balance between computational efficiency and detection performance. RTUAV-YOLO achieves an average improvement of 3.4% and 2.4% in mAP50 and mAP50-95, respectively, compared to the baseline model YOLOv11, while reducing the number of parameters by 65.3%. Furthermore, cross-dataset evaluations on the UAVDT and UAVVaste benchmarks confirm RTUAV-YOLO’s generalization and robustness across di-verse UAV aerial imagery conditions. When deployed on the Jetson Orin Nano, a common platform for UAVs, our method achieves a real-time detection frame rate of 37.8 FPS while maintaining excellent detection accuracy, providing an effective solution for real-time target detection on real-world UAVs.

Although this investigation has yielded promising outcomes, opportunities remain for further computational complexity reduction to facilitate deployment on ultra-low-power UAV platforms. Future research directions will encompass the development and implementation of advanced lightweight architectural components and optimized loss functions to further minimize computational requirements while enhancing detection accuracy and robustness in challenging scenarios involving high object density and severe occlusion conditions.

## Figures and Tables

**Table 1:** Experimental comparison with YOLO series models on VisDrone2019.

Model	P (%)	R (%)	mAP50 (%)	mAP50-95 (%)	Params (M)	FLOPs (G)	FPS
YOLOv8-N	42.5	33.9	33.3	19.6	3.16	8.9	184
YOLOv8-S	47.9	37.8	39.1	23.4	11.17	28.8	143
YOLOv8-M	53.7	43.2	44.4	27.1	25.90	79.3	89
YOLOv8-L	56.7	44.1	45.9	28.4	43.69	165.7	66
YOLOv8-X	57.5	44.6	46.8	28.9	68.23	258.5	48
YOLOv11-N	42.3	33.6	33.1	19.3	2.62	6.6	192
YOLOv11-S	48.2	38.1	39.4	23.6	9.46	21.7	160
YOLOv11-M	53.4	43.0	44.1	26.9	20.11	68.5	97
YOLOv11-L	56.9	44.3	46.0	28.5	25.37	87.6	75
YOLOv11-X	57.7	44.8	46.9	29.0	56.97	196.0	56
RTUAV-YOLO-N	44.7	35.6	35.9	21.3	0.79	8.3	187
RTUAV-YOLO-S	52.5	41.1	42.9	25.9	2.49	21.4	162
RTUAV-YOLO-M	57.5	44.7	46.8	28.9	7.23	56.1	109
RTUAV-YOLO-L	60.2	46.0	49.2	30.5	10.43	79.1	88
RTUAV-YOLO-X	61.3	48.1	51.4	32.4	22.86	168.1	65

**Table 2:** Experimental comparison with SOTA UAV object detection models on VisDrone2019.

Model	mAP50 (%)	mAP50-95 (%)	Params (M)	FLOPs (G)	FPS
PS-YOLO-N	33.2	19.3	1.39	5.1	213
PS-YOLO-S	40.7	24.2	5.53	20.0	178
PS-YOLO-M	46.0	28.3	14.82	73.5	91
MUSD-YOLO	43.4	-	6.77	-	82
LGFF-YOLO	43.5	22.9	4.15	12.4	181
RT-DETR-R18	44.6	26.7	20.03	60.0	101
RT-DETR-R34	46.0	27.2	31.04	92.0	78
RT-DETR-R50	48.3	28.8	42.02	136.0	69
FBRT-YOLO-N	34.4	20.2	0.94	6.7	192
FBRT-YOLO-S	42.4	25.9	2.92	22.9	143
FBRT-YOLO-M	45.9	28.4	7.26	58.7	94
FBRT-YOLO-L	47.7	29.7	14.63	119.2	70
FBRT-YOLO-X	48.4	30.1	22.89	185.8	52
CSFPR-RTDETR	42.3	24.9	14.09	63.9	98
UAV-DETR-EV2	47.5	28.7	13.02	43.1	101
UAV-DETR-R18	48.8	29.8	20.19	70.2	91
UAV-DETR-R50	51.1	31.5	42.07	170.6	61
RTUAV-YOLO-N	35.9	21.3	0.79	8.3	187
RTUAV-YOLO-S	42.9	25.9	2.49	21.4	162
RTUAV-YOLO-M	46.8	28.9	7.23	56.1	109
RTUAV-YOLO-L	49.2	30.5	10.43	79.1	88
RTUAV-YOLO-X	51.4	32.4	22.86	168.1	65

**Table 3:** Experimental results on the UAVDT dataset.

Model	mAP50 (%)	mAP75 (%)	mAP50-95 (%)
YOLOv11-X [11]	28.7	16.8	15.8
ClusDet [51]	26.5	12.5	13.7
GLSAN [52]	28.1	18.8	17.0
GFL [53]	29.5	17.9	16.9
CEASC [54]	30.9	17.8	17.1
FBRT-YOLO [47]	31.1	18.9	18.4
RTUAV-YOLO-X	31.8	19.3	18.8

**Table 4:** Experimental results on the UAVVaste dataset.

Model	mAP50 (%)	mAP75 (%)	mAP50-95 (%)
YOLOv11-S [11]	63.0	41.2	27.8
HIC-YOLOv5 [55]	65.1	43.1	30.5
RT-DETR-R18 [48]	72.6	45.7	36.3
RT-DETR-R50 [48]	73.5	47.1	37.4
RTUAV-YOLO-X	76.8	49.7	38.2

**Table 5:** Ablation experiment results. × indicates that the method was not used, and √ indicates that the method was used.

Backbone	LDSM	MSFAM	PDSCM	MPDWIoU	mAP50 (%)	mAP50-95 (%)	Params (M)	FLOPs (G)
×	×	×	×	×	39.4	23.6	9.46	21.7
√	×	×	×	×	39.9	23.9	3.87	27.0
√	√	×	×	×	40.1	24.0	3.22	23.7
√	×	√	×	×	40.5	24.6	3.62	25.7
√	×	×	√	×	40.4	24.3	3.56	25.4
√	×	×	×	√	40.3	24.2	3.87	27.0
√	√	×	√	×	41.4	24.8	3.01	24.2
√	×	√	√	×	41.6	24.9	3.12	23.9
√	√	√	×	×	41.8	25.1	2.94	22.9
√	√	√	√	×	42.4	25.5	2.49	21.4
√	√	√	√	√	42.9	25.9	2.49	21.4

**Table 6:** Comparison of different IoU-based loss functions using RTUAV-YOLO-S.

Loss Function	mAP50 (%)	mAP50-95 (%)	Params (M)	FLOPs (G)
GIoU [34]	42.3	25.5	2.49	21.4
DIoU [35]	42.2	25.2	2.49	21.4
CIoU [36]	42.4	25.5	2.49	21.4
WIoUv3 [37]	42.5	25.6	2.49	21.4
MPDWIoU (Ours)	42.9	25.9	2.49	21.4

**Table 7:** Quantitative evaluation results on Jetson Orin Nano.

Model	mAP50 (%)	mAP50-95 (%)	Params (M)	FPS
YOLOv8-N	33.3	19.6	3.16	35.2
YOLOv8-S	39.1	23.4	11.17	20.1
YOLOv11-N	33.1	19.3	2.62	45.1
YOLOv11-S	39.4	23.6	9.46	23.3
PS-YOLO-N	33.2	19.3	1.39	41.1
PS-YOLO-S	40.7	24.2	5.53	26.9
FBRT-YOLO-N	34.4	20.2	0.94	52.6
FBRT-YOLO-S	42.4	25.9	2.92	34.1
UAV-DETR-EV2	47.5	28.7	13.02	19.4
RTUAV-YOLO-N	35.9	21.3	0.79	53.7
RTUAV-YOLO-S	42.9	25.9	2.49	37.8

**Table 8:** Comparative experimental results of the baseline model and RTUAV-YOLO on the HIT-UAV dataset.

Model	mAP50 (%)	mAP50-95 (%)	Params (M)	FLOPs (G)
YOLOv8-N	71.4	43.2	3.16	8.9
YOLOv8-S	80.1	49.3	11.17	28.8
YOLOv8-M	86.2	54.2	25.90	79.3
YOLOv11-N	71.7	43.2	2.62	6.6
YOLOv11-S	80.6	49.5	9.46	21.7
YOLOv11-M	87.1	54.8	20.11	68.5
RTUAV-YOLO-N	76.9	43.2	0.79	8.3
RTUAV-YOLO-S	82.3	49.3	2.49	21.4
RTUAV-YOLO-M	89.2	57.1	7.23	56.1

**Table 9:** Comparative experimental results of the baseline model and RTUAV-YOLO on the AI-TOD dataset.

Model	mAP50 (%)	mAP50-95 (%)	Params (M)	FLOPs (G)
YOLOv8-N	39.2	17.5	3.16	8.9
YOLOv8-S	43.6	19.1	11.17	28.8
YOLOv8-M	46.1	22.3	25.90	79.3
YOLOv11-N	39.6	17.9	2.62	6.6
YOLOv11-S	43.9	19.2	9.46	21.7
YOLOv11-M	46.7	22.8	20.11	68.5
RTUAV-YOLO-N	40.1	18.6	0.79	8.3
RTUAV-YOLO-S	46.1	20.8	2.49	21.4
RTUAV-YOLO-M	49.2	25.3	7.23	56.1

## Data Availability

The data that support the findings of this study are available from the corresponding author upon reasonable request. The project page: https://gitee.com/zhangruizhi0110/RTUAV-YOLO.

## References

[1] Liu X., He J., Yao Y., Zhang J., Liang H., Wang H., Hong Y. (2017). Classifying Urban Land Use by Integrating Remote Sensing and Social Media Data. Int. J. Geogr. Inf. Sci..

[2] Byun S., Shin I.-K., Moon J., Kang J., Choi S.-I. (2021). Road Traffic Monitoring from UAV Images Using Deep Learning Networks. Remote Sens..

[3] Cao D., Zhang B., Zhang X., Yin L., Man X. (2023). Optimization Methods on Dynamic Monitoring of Mineral Reserves for Open Pit Mine Based on UAV Oblique Photogrammetry. Measurement.

[4] Albattah W., Masood M., Javed A., Nawaz M., Albahli S. (2023). Custom CornerNet: A Drone-Based Improved Deep Learning Technique for Large-Scale Multiclass Pest Localization and Classification. Complex Intell. Syst..

[5] Zhang H., Wang L., Tian T., Yin J. (2021). A Review of Unmanned Aerial Vehicle Low-Altitude Remote Sensing (UAV-LARS) Use in Agricultural Monitoring in China. Remote Sens..

[6] Sun G., He L., Sun Z., Wu Q., Liang S., Li J., Niyato D., Leung V.C.M. (2024). Joint Task Offloading and Resource Allocation in Aerial-Terrestrial UAV Networks With Edge and Fog Computing for Post-Disaster Rescue. IEEE Trans. Mob. Comput..

[7] Teixidó P., Gómez-Galán J.A., Caballero R., Pérez-Grau F.J., Hinojo-Montero J.M., Muñoz-Chavero F., Aponte J. (2021). Secured Perimeter with Electromagnetic Detection and Tracking with Drone Embedded and Static Cameras. Sensors.

[8] Xie X., Cheng G., Wang J., Yao X., Han J. Oriented R-CNN for Object Detection. Proceedings of the IEEE/CVF International Conference on Computer Vision (ICCV).

[9] Ren S., He K., Girshick R., Sun J. (2017). Faster R-CNN: Towards Real-Time Object Detection with Region Proposal Networks. IEEE Trans. Pattern Anal. Mach. Intell..

[10] Liu W., Anguelov D., Erhan D., Szegedy C., Reed S., Fu C.-Y., Berg A.C., Leibe B., Matas J., Sebe N., Welling M. (2016). SSD: Single Shot MultiBox Detector. Proceedings of the Computer Vision—ECCV 2016.

[11] Redmon J., Divvala S., Girshick R., Farhadi A. You Only Look Once: Unified, Real-Time Object Detection. Proceedings of the IEEE Conference on Computer Vision and Pattern Recognition (CVPR).

[12] Weng S., Wang H., Wang J., Xu C., Zhang E. (2025). YOLO-SRMX: A Lightweight Model for Real-Time Object Detection on Unmanned Aerial Vehicles. Remote Sens..

[13] Liu Y., He M., Hui B. (2025). ESO-DETR: An Improved Real-Time Detection Transformer Model for Enhanced Small Object Detection in UAV Imagery. Drones.

[14] Luo X., Zhu X. (2025). YOLO-SMUG: An Efficient and Lightweight Infrared Object Detection Model for Unmanned Aerial Vehicles. Drones.

[15] He K., Zhang X., Ren S., Sun J. (2015). Spatial Pyramid Pooling in Deep Convolutional Networks for Visual Recognition. IEEE Trans. Pattern Anal. Mach. Intell..

[16] Girshick R. Fast R-CNN. Proceedings of the IEEE International Conference on Computer Vision.

[17] Khanam R., Hussain M. (2024). YOLOv11: An Overview of the Key Architectural Enhancements. arXiv.

[18] Lin T.-Y., Maire M., Belongie S., Hays J., Perona P., Ramanan D., Dollár P., Zitnick C.L., Fleet D., Pajdla T., Schiele B., Tuytelaars T. (2014). Microsoft COCO: Common Objects in Context. Proceedings of the Computer Vision—ECCV 2014.

[19] Li Y., Li Q., Pan J., Zhou Y., Zhu H., Wei H., Liu C. (2024). SOD-YOLO: Small-Object-Detection Algorithm Based on Improved YOLOv8 for UAV Images. Remote Sens..

[20] Zhou S., Zhou H., Qian L. (2025). A Multi-Scale Small Object Detection Algorithm SMA-YOLO for UAV Remote Sensing Images. Sci. Rep..

[21] Guo C., Fan B., Zhang Q., Xiang S., Pan C. AugFPN: Improving Multi-Scale Feature Learning for Object Detection. Proceedings of the IEEE/CVF Conference on Computer Vision and Pattern Recognition (CVPR).

[22] Cheng G., Lang C., Wu M., Xie X., Yao X., Han J. (2021). Feature Enhancement Network for Object Detection in Optical Remote Sensing Images. J. Remote Sens..

[23] Zhang K., Shen H. (2022). Multi-Stage Feature Enhancement Pyramid Network for Detecting Objects in Optical Remote Sensing Images. Remote Sens..

[24] Li H., Li Y., Xiao L., Zhang Y., Cao L., Wu D. (2025). RLRD-YOLO: An Improved YOLOv8 Algorithm for Small Object Detection from an Unmanned Aerial Vehicle (UAV) Perspective. Drones.

[25] Chang J., Lu Y., Xue P., Xu Y., Wei Z. (2022). Automatic Channel Pruning via Clustering and Swarm Intelligence Optimization for CNN. Appl. Intell..

[26] Guo S., Wang Y., Li Q., Yan J. DMCP: Differentiable Markov Channel Pruning for Neural Networks. Proceedings of the IEEE/CVF Conference on Computer Vision and Pattern Recognition (CVPR).

[27] He Y., Zhang X., Sun J. Channel Pruning for Accelerating Very Deep Neural Networks. Proceedings of the IEEE International Conference on Computer Vision (ICCV).

[28] Liu Z., Li J., Shen Z., Huang G., Yan S., Zhang C. Learning Efficient Convolutional Networks Through Network Slimming. Proceedings of the IEEE International Conference on Computer Vision (ICCV).

[29] Howard A.G., Zhu M., Chen B., Kalenichenko D., Wang W., Weyand T., Andreetto M., Adam H. (2017). MobileNets: Efficient Convolutional Neural Networks for Mobile Vision Applications. arXiv.

[30] Zhang X., Zhou X., Lin M., Sun J. ShuffleNet: An Extremely Efficient Convolutional Neural Network for Mobile Devices. Proceedings of the IEEE Conference on Computer Vision and Pattern Recognition (CVPR).

[31] Han K., Wang Y., Tian Q., Guo J., Xu C., Xu C. GhostNet: More Features From Cheap Operations. Proceedings of the IEEE/CVF Conference on Computer Vision and Pattern Recognition (CVPR).

[32] Fan Q., Li Y., Deveci M., Zhong K., Kadry S. (2025). LUD-YOLO: A Novel Lightweight Object Detection Network for Unmanned Aerial Vehicle. Inf. Sci..

[33] Cao J., Bao W., Shang H., Yuan M., Cheng Q. (2023). GCL-YOLO: A GhostConv-Based Lightweight YOLO Network for UAV Small Object Detection. Remote Sens..

[34] Rezatofighi H., Tsoi N., Gwak J., Sadeghian A., Reid I., Savarese S. Generalized Intersection Over Union: A Metric and a Loss for Bounding Box Regression. Proceedings of the IEEE/CVF Conference on Computer Vision and Pattern Recognition (CVPR).

[35] Zheng Z., Wang P., Liu W., Li J., Ye R., Ren D. Distance-IoU Loss: Faster and Better Learning for Bounding Box Regression. Proceedings of the AAAI Conference on Artificial Intelligence.

[36] Zheng Z., Wang P., Ren D., Liu W., Ye R., Hu Q., Zuo W. (2020). Enhancing Geometric Factors in Model Learning and Inference for Object Detection and Instance Segmentation. arXiv.

[37] Tong Z., Chen Y., Xu Z., Yu R. (2023). Wise-IoU: Bounding Box Regression Loss with Dynamic Focusing Mechanism. arXiv.

[38] Zhang Y.-F., Ren W., Zhang Z., Jia Z., Wang L., Tan T. (2022). Focal and Efficient IOU Loss for Accurate Bounding Box Regression. Neurocomputing.

[39] Lu S., Lu H., Dong J., Wu S. (2023). Object Detection for UAV Aerial Scenarios Based on Vectorized IOU. Sensors.

[40] Du D., Zhu P., Wen L., Bian X., Lin H., Hu Q., Peng T., Zheng J., Wang X., Zhang Y. VisDrone-DET2019: The Vision Meets Drone Object Detection in Image Challenge Results. Proceedings of the IEEE/CVF International Conference on Computer Vision (ICCV).

[41] Du D., Qi Y., Yu H., Yang Y., Duan K., Li G., Zhang W., Huang Q., Tian Q. The Unmanned Aerial Vehicle Benchmark: Object Detection and Tracking. Proceedings of the European Conference on Computer Vision (ECCV).

[42] Kraft M., Piechocki M., Ptak B., Walas K. (2021). Autonomous, Onboard Vision-Based Trash and Litter Detection in Low Altitude Aerial Images Collected by an Unmanned Aerial Vehicle. Remote Sens..

[43] Jocher G., Chaurasia A., Qiu J. (2023). YOLOv8 by Ultralytics. https://github.com/ultralytics/ultralytics.

[44] Zhong H., Zhang Y., Shi Z., Zhang Y., Zhao L. (2025). PS-YOLO: A Lighter and Faster Network for UAV Object Detection. Remote Sens..

[45] Zhao X., Zhang H., Zhang W., Ma J., Li C., Ding Y., Zhang Z. (2025). MSUD-YOLO: A Novel Multiscale Small Object Detection Model for UAV Aerial Images. Drones.

[46] Peng H., Xie H., Liu H., Guan X. (2024). LGFF-YOLO: Small Object Detection Method of UAV Images Based on Efficient Local–Global Feature Fusion. J. Real-Time Image Proc..

[47] Xiao Y., Xu T., Xin Y., Li J. FBRT-YOLO: Faster and Better for Real-Time Aerial Image Detection. Proceedings of the AAAI Conference on Artificial Intelligence.

[48] Zhao Y., Lv W., Xu S., Wei J., Wang G., Dang Q., Liu Y., Chen J. DETRs Beat YOLOs on Real-Time Object Detection. Proceedings of the IEEE/CVF Conference on Computer Vision and Pattern Recognition (CVPR).

[49] Hu L., Yuan J., Cheng B., Xu Q. (2025). CSFPR-RTDETR: Real-Time Small Object Detection Network for UAV Images Based on Cross-Spatial-Frequency Domain and Position Relation. IEEE Trans. Geosci. Remote Sens..

[50] Zhang H., Liu K., Gan Z., Zhu G.-N. (2025). UAV-DETR: Efficient End-to-End Object Detection for Unmanned Aerial Vehicle Imagery. arXiv.

[51] Yang F., Fan H., Chu P., Blasch E., Ling H. Clustered Object Detection in Aerial Images. Proceedings of the IEEE/CVF International Conference on Computer Vision (ICCV).

[52] Deng S., Li S., Xie K., Song W., Liao X., Hao A., Qin H. (2021). A Global-Local Self-Adaptive Network for Drone-View Object Detection. IEEE Trans. Image Process..

[53] Li X., Wang W., Wu L., Chen S., Hu X., Li J., Tang J., Yang J. (2020). Generalized Focal Loss: Learning Qualified and Distributed Bounding Boxes for Dense Object Detection. Proceedings of the Advances in Neural Information Processing Systems.

[54] Du B., Huang Y., Chen J., Huang D. Adaptive Sparse Convolutional Networks With Global Context Enhancement for Faster Object Detection on Drone Images. Proceedings of the IEEE/CVF Conference on Computer Vision and Pattern Recognition (CVPR).

[55] Tang S., Zhang S., Fang Y. HIC-YOLOv5: Improved YOLOv5 For Small Object Detection. Proceedings of the 2024 IEEE International Conference on Robotics and Automation (ICRA).

[56] Suo J., Wang T., Zhang X., Chen H., Zhou W., Shi W. (2023). HIT-UAV: A High-Altitude Infrared Thermal Dataset for Unmanned Aerial Vehicle-Based Object Detection. Sci. Data.

[57] Wang J., Yang W., Guo H., Zhang R., Xia G.-S. Tiny Object Detection in Aerial Images. Proceedings of the 2020 25th International Conference on Pattern Recognition (ICPR).

